# Exploratory longitudinal monitoring of physiological, hematological, psychological, and irisin responses during an 8-week pre-competition period in elite rhythmic gymnasts

**DOI:** 10.3389/fspor.2026.1925319

**Published:** 2026-10-21

**Authors:** Min Ye, Yu Qian, Shuang Ren, Johanna Kéringer, Chenxin Lin, Mengying Wang, Zsolt Radák

**Affiliations:** 1 Research Center for Molecular Exercise Science, Doctoral School of Sport Sciences, Hungarian University of Sports Science, Budapest, Hungary; 2 Department of Sports Management, Hungarian University of Sports Science, Budapest, Hungary; 3 Chengdu Sport University, Chengdu, China

**Keywords:** exploratory study, hematological monitoring, irisin, multidimensional monitoring, psychological stress, rhythmic gymnastics, training load monitoring

## Abstract

**Introduction:**

Training load monitoring has been widely studied across many sports, yet evidence in rhythmic gymnastics remains limited despite its high energetic demands, technical complexity, and strict body composition requirements. This exploratory pilot study examined physiological, hematological, perceptual, psychological, and irisin responses during an 8-week pre-competition period in eight high-level female rhythmic gymnasts.

**Methods:**

A multidimensional monitoring approach included heart rate assessment, lipid profiling, urinalysis, complete blood count, body composition analysis, rating of perceived exertion (RPE), and the Chinese-language Athlete Training State Monitoring Scale developed by Yan.

**Results:**

Irisin concentrations increased over the training period (nominal *p* = 0.033), while mean corpuscular hemoglobin concentration remained below reference ranges. Exploratory associations were observed between changes in irisin and selected psychological variables, including a strong negative association with individual mean emotional stress across the monitoring period (*r* = −0.859, nominal *p* = 0.006) and a positive association with individual mean psychological exhaustion (*r* = 0.704, nominal *p* = 0.051). Several psychological outcomes showed no clear evidence of longitudinal change.

**Discussion:**

These findings suggest that multidimensional monitoring may provide useful insight into the interaction between physiological adaptation and perceived recovery status during intensified pre-competition training in high-level rhythmic gymnasts. Given the small sample size and exploratory design, these findings should be interpreted cautiously.

## 1 Introduction

Rhythmic gymnastics is characterized by exceptional technical complexity, artistic precision, and high physiological and energetic demands. Under the current International Gymnastics Federation Code of Points (2025–2028) (1), there is no upper limit for difficulty scores, incentivizing athletes to continuously increase the intensity and complexity of their routines. Such demands require substantial physiological adaptation while simultaneously imposing considerable psychological stress. Previous research has demonstrated that performance in rhythmic gymnastics is strongly influenced by physiological and anthropometric characteristics (2). Longitudinal monitoring in elite rhythmic gymnasts has revealed marked variations in training load and recovery status across competitive seasons (3), and psychological resilience has been identified as a critical factor in managing stress and injury risk (4). Consequently, systematic monitoring of training load is essential to optimize performance while minimizing the risk of overtraining and injury.

Training load monitoring strategies are commonly categorized into internal and external measures (5). Internal load can be assessed using subjective tools such as the Rating of Perceived Exertion (6) and the Recovery-Stress Questionnaire for Athletes (7), whereas external load is typically quantified through performance and biomechanical metrics, including training volume, session duration, and biomechanical workload characteristics (8). Excessive training without adequate recovery may lead to chronic fatigue and underperformance syndrome (9). Although monitoring approaches have been extensively investigated across endurance and team sports, including water polo (10), cycling (11), soccer (12), basketball (13), golf (14), and football (15), rhythmic gymnastics remains comparatively under-explored, particularly regarding integrated physiological, hematological, perceptual, and psychological monitoring frameworks. Recent reviews further emphasize the importance of physical fitness parameters in rhythmic gymnastics but highlight the need for more applied longitudinal data in elite populations (16).

In elite aesthetic sports such as rhythmic gymnastics, training adaptation is likely influenced by the interaction of physiological load, recovery capacity, metabolic regulation, hematological status, and psychological stress. Consequently, monitoring approaches based on a single physiological marker may not adequately capture the multidimensional nature of adaptation during intensified training periods. A longitudinal monitoring framework that integrates physiological, hematological, psychological, and perceptual measures may therefore provide a more ecologically valid understanding of athlete responses during pre-competition preparation.

Irisin, an exercise-responsive myokine primarily secreted by skeletal muscle, has attracted growing attention as a potential marker associated with exercise adaptation and metabolic regulation (17). Previous studies have implicated irisin in exercise-related energy metabolism and adipose tissue browning (18), skeletal muscle remodeling (19), and exercise-mediated cardiovascular protection (20). Emerging evidence has also reported relationships between circulating irisin concentrations and selected psychological outcomes, including fatigue and depressive symptoms (21, 22). However, these associations remain incompletely understood, particularly in elite athletes and aesthetic sports settings. Therefore, in the present study, irisin was included as an exploratory exercise-responsive biomarker within a broader multidimensional monitoring framework rather than as a definitive mechanistic marker of psychological adaptation.

Importantly, it remains unclear how physiological and psychological responses interact during intensified pre-competition training in aesthetic sports. Understanding these multidimensional responses may contribute to improved athlete monitoring and training management in high-level rhythmic gymnasts. Therefore, the aim of the present exploratory pilot study was to examine longitudinal physiological, hematological, perceptual, psychological, and irisin responses during an 8-week pre-competition period in elite rhythmic gymnasts using a multidimensional monitoring approach. We hypothesized that intensified pre-competition training would be associated with measurable multidimensional responses across physiological, hematological, perceptual, and psychological domains. Given the exploratory nature of the study, no specific mechanistic hypothesis regarding irisin-mediated psychological adaptation was proposed.

## 2 Methods

### 2.1 Ethical approval

The study protocol was approved by the Human Ethics Committee of Chengdu Sport University (Approval No. 2021-60) and was conducted in accordance with the Declaration of Helsinki. All participants received detailed oral and written information regarding the study procedures and provided written informed consent prior to participation. The informed-consent documentation permitted the use of participants' data for research purposes under conditions of confidentiality and specified that participants' identities would not be disclosed in publications. One participant was 17 years old at enrollment and was a professional athlete whose principal means of support was her own employment income. Under Article 18 of the Civil Code of the People's Republic of China, individuals aged 16 years or older whose principal means of support is their own employment income are regarded as having full capacity for civil conduct.

### 2.2 Participants and setting

Eight highly trained national-level female rhythmic gymnasts from a high-level competitive team in China participated in this study during an 8-week pre-competition preparation period. All eight athletes held a nationally recognized high-level athlete classification in China and competed at the highest level of national competition. According to the Participant Classification Framework proposed by McKay et al. (23), the participants were classified as Tier 3 (Highly Trained/National Level), based on their nationally recognized athlete classification, structured high-performance training, and participation at the highest level of domestic competition in China. To minimize the risk of indirect identification, specific information regarding the team and competition results is not reported.

The study cohort comprised the eight athletes participating in the monitored pre-competition training program. No additional selection based on physiological, hematological, body-composition, or psychological characteristics was applied. The sample size was determined by the number of athletes available in the monitored training squad during the study period; therefore, no formal *a priori* sample-size calculation was performed.

The pre-competition training period commenced in July and lasted eight weeks (Figure 1). Participant characteristics are presented in Table 1.

**Figure 1:**
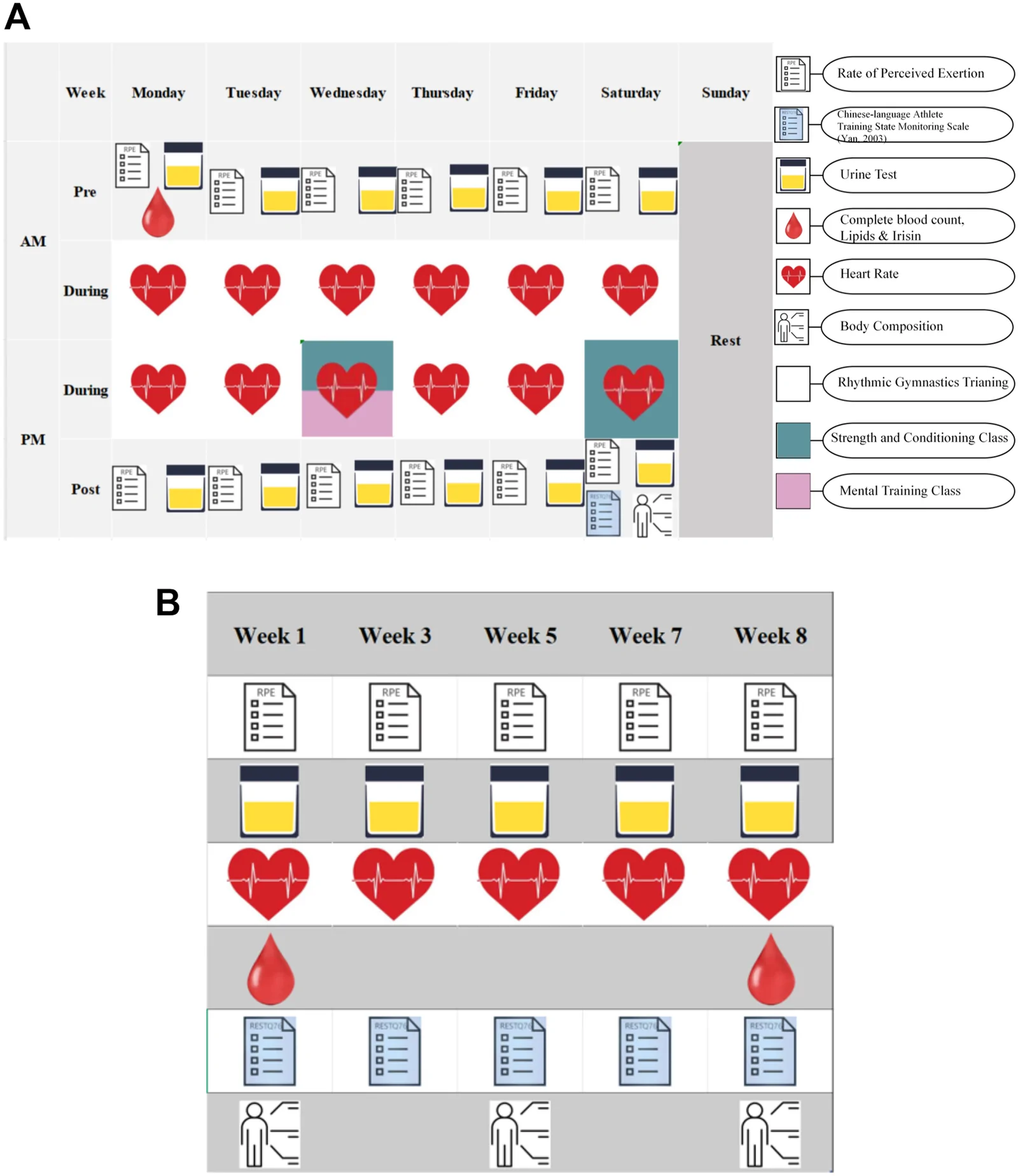
Experimental design and data collection protocol. **(A)** Overview of the 8-week pre-competition training and monitoring protocol. The training program consisted of morning and afternoon training sessions, including rhythmic gymnastics training, strength and conditioning, psychological preparation, and recovery activities. **(B)** Timeline of longitudinal assessments. RPE, urine testing, heart rate monitoring, and Chinese-language Athlete Training State Monitoring Scale (1) assessments were conducted on designated monitoring days at Weeks 1, 3, 5, 7, and 8. Blood samples were collected at Weeks 1 and 8, whereas body composition was assessed at Weeks 1, 5, and 8.

**Table 1:** Characteristics of the gymnasts.

Number (*n*)	Age (years)	Training years	Height (cm)	Weight (kg)
8	19 ± 1.31	11.75 ± 1.67	167.45 ± 3.90	49.66 ± 2.99

Values are presented as mean ± standard deviation (SD) unless otherwise indicated. Parameters include the number of gymnasts (*n*), age (years), training experience (years), height (cm), and weight (kg).

#### 2.2.1 Training program

The pre-competition mesocycle consisted of six training days per week, with two daily training sessions (morning and afternoon). Each session was scheduled to last approximately 4 h; however, actual training duration could vary depending on the completion of technical tasks and daily training requirements. The training program integrated flexibility training, apparatus-specific technical preparation, jump and balance sequences, routine repetitions, strength and conditioning sessions, psychological skills training, and recovery-oriented stretching, with progressive emphasis on routine execution quality and competition-specific success criteria (Tables 2, 3).

**Table 2:** Representative weekly training structure during the 8-week pre-competition mesocycle.

Day	Morning session	Afternoon session
Monday	Flexibility training; shoulder and trunk mobility drills; ball-based core exercises; traveling kick sequences integrated with routines; five-ball apparatus handling and routine segment practice; full routine repetitions with predefined successful execution criteria	Flexibility training; balance-pad control drills; hoop and clubs apparatus sequences; apparatus throw-and-catch combinations; segmented and full routine practice emphasizing high-quality execution
Tuesday	Flexibility and floor exercises; apparatus-specific jump sequences; five-ball technical combinations; segmented routine practice and full routine repetitions	Flexibility and balance-control exercises; hoop and clubs jump sequences; apparatus handling drills; segmented routine repetitions and full routine execution
Wednesday	Flexibility; trunk rotation sequences; ball-based core and lateral trunk exercises; jump combinations; apparatus-specific free technical activity and routine repetitions	Psychological skills training; strength and conditioning session
Thursday	Flexibility; balance and kick-control exercises; clubs apparatus difficulty sequences; segmented and combined apparatus routine execution with predefined success targets	Flexibility; floor exercises; jump sequences; additional technical repetition for low-success elements; reduced-difficulty routine execution
Friday	Flexibility; balance-control drills; hoop and clubs technical sequences; targeted repetition of successful apparatus combinations; modified routine execution	Flexibility; ball-based core exercises; integrated routine movement sequences; apparatus-specific activity; high-quality routine execution for both five-ball and hoop/clubs routines
Saturday	Flexibility; balance-control exercises; jump sequences; apparatus activity and rotational full-routine execution across multiple competition areas	Strength and conditioning session
Sunday	Recovery day	Recovery day

This table summarizes the representative weekly training structure implemented during the 8-week pre-competition preparation period in elite rhythmic gymnasts. Training sessions were organized into morning and afternoon blocks across six training days per week, with Sunday designated as a recovery day. The training program included flexibility training, apparatus-specific technical preparation, jump and balance sequences, routine repetitions, strength and conditioning sessions, psychological skills training, and recovery-oriented stretching. Technical training emphasized progressive routine execution quality and predefined success criteria for apparatus handling and routine completion.

**Table 3:** Characteristics of the pre-competition training mesocycle.

Variable	Description
Training frequency	6 days/week
Rest day	Sunday
Training structure	Morning and afternoon sessions
Scheduled training duration	Approximately 4 h per session (actual duration varied according to completion of technical tasks and training requirements)
Main technical content	Apparatus handling, jump sequences, balance and flexibility drills, routine repetitions
Apparatus	Five-ball, hoop, clubs
Conditioning training	Twice weekly
Psychological training	Once weekly
Recovery strategy	Daily stretching and flexibility-based cool-down
Monitoring methods	HR, RPE, Chinese-language Athlete Training State Monitoring Scale (1), blood analysis, urinalysis, body composition

This table summarizes the characteristics of the 8-week pre-competition training mesocycle and the multidimensional monitoring framework used in the present exploratory study. Parameters include training frequency, training structure, rhythmic gymnastics-specific technical preparation, apparatus handling, jump and balance sequences, conditioning sessions, recovery strategies, and monitoring methods. Monitoring variables included heart rate assessment, rating of perceived exertion (RPE), the Chinese-language Athlete Training State Monitoring Scale (1), blood analysis, urinalysis, and body composition measurements.

The pre-competition mesocycle was characterized by progressive technical refinement and competition-specific preparation. Training intensity distribution was monitored using heart-rate recordings during designated monitoring weeks throughout the intervention period. Internal load monitoring included RPE and Chinese-language Athlete Training State Monitoring Scale (24) assessments, while physiological monitoring included blood analysis, urinalysis, and body composition assessment.

Although the overall training structure, frequency, scheduled duration, and technical contents were systematically documented, detailed quantitative external-load variables, including total apparatus repetitions, jump counts, landing counts, and biomechanical workload, were not systematically recorded. Therefore, these factors should be considered limitations of the present exploratory monitoring approach.

### 2.3 Measures

#### 2.3.1 Physiological indicators

Blood samples were collected on Monday mornings during Weeks 1 and 8. Two 5 mL venous blood samples were obtained from each gymnast in a fasted state for complete blood count (CBC), lipid profile, and irisin analyses. Participants were instructed to maintain their habitual nutritional and hydration routines prior to each assessment. However, menstrual cycle phase, detailed dietary intake, sleep quality, and psychosocial stress outside sport were not systematically controlled and were therefore considered potential confounding factors.

**CBC and lipid profile** analyses were performed at the Department of Clinical Laboratory, Affiliated Sports Hospital of Chengdu Sport University.

**Serum irisin** concentrations were measured using a commercially available enzyme-linked immunosorbent assay (ELISA) kit (Elabscience; Wuhan E-Lyte Biotechnology Co., Ltd., Wuhan, China) according to the manufacturer's instructions. The exact catalogue and lot numbers of the ELISA kit could not be recovered from the retained laboratory records. Whole-blood samples were allowed to stand at room temperature for 1 h and were then centrifuged at 1,000 × g for 20 min to obtain serum. Serum samples were analyzed in duplicate after a 2-fold dilution with the sample diluent provided by the manufacturer. Briefly, 100 μL of each diluted serum sample was added to the designated wells and incubated at 37 °C for 90 min. Subsequently, 100 μL of biotinylated detection antibody was added and incubated at 37 °C for 60 min. After three washing cycles, 100 μL of enzyme-conjugate working solution was added and incubated at 37 °C for 30 min. The plate was then washed five times, followed by the addition of 90 μL of TMB substrate and incubation at 37 °C in the dark for approximately 15 min. The reaction was terminated by adding 50 μL of stop solution, and optical density was immediately measured at 450 nm using a microplate reader.

The manufacturer-provided calibrator range was 78.13–5,000 pg/mL. After accounting for the 1:2 serum dilution, this corresponded to a serum-equivalent calibration range of 156.25–10,000 pg/mL. Calibration standards were assayed as single wells, whereas serum samples were analyzed in duplicate. The manufacturer's instructions permitted polynomial curve fitting for calibration, and the surviving original assay calculation record documented the use of a second-order polynomial model to convert blank-corrected OD values to on-plate irisin concentrations. The calibration equation was *y* = 395.8*x*^2^ + 1,014.3*x* + 42.208, where *x* represents the blank-corrected OD and *y* represents the on-plate concentration (pg/mL), with *R*^2^ = 0.9994. All Week 1 and Week 8 serum samples were analyzed on the same ELISA plate and quantified using the same calibration relationship. On-plate concentrations were multiplied by two to account for the 1:2 sample dilution and obtain the corresponding serum concentrations. All individual on-plate sample concentrations fell within the calibrator range; therefore, no sample concentration was derived by extrapolation beyond the calibration range.

All serum samples were analyzed in duplicate. The coefficient of variation (CV) between replicate OD measurements was calculated as a descriptive measure of within-pair agreement. No prospectively predefined numerical duplicate-CV exclusion threshold was applied. Individual replicate OD values and corresponding CVs are reported in Supplementary Table S2. The observed duplicate CVs ranged from 5.87% to 13.15%, and no replicate was excluded on the basis of duplicate CV. The mean of the two replicate OD measurements was used for concentration calculation. Because OD-B was consistently higher than OD-A across all 16 sample pairs, the between-replicate differences were not interpreted as reflecting random analytical imprecision alone. The available historical records did not permit retrospective determination of the source of this directional difference; therefore, a systematic positional, pipetting-order, reading-order, or other technical effect cannot be excluded.

**Urine samples** were collected approximately 15 min before the morning training session and approximately 15 min after the afternoon training session. The two sampling conditions were therefore defined as Pre-training and Post-training. Monitoring weeks were assigned according to the actual sampling dates.

For each athlete, valid observations obtained within the same monitoring week and sampling condition were averaged to generate one athlete-level weekly value. Missing observations were retained as missing and were excluded from the calculation of the corresponding weekly mean; no missing value was replaced by zero. For urine specific gravity (SG), readings reported as <1.005 and >1.030 were converted to the corresponding reporting-boundary values of 1.005 and 1.030, respectively. The other quantitative or semi-quantitative urinalysis variables were converted according to the predefined coding scheme used for the test-strip reporting categories.

The final athlete-level weekly data were analyzed using a two-way repeated-measures analysis of variance with Week and sampling Condition (Pre-training vs. Post-training) as within-subject factors. Both factors were matched within subject, and the full model including the Week × Condition interaction was fitted. Geisser–Greenhouse correction was applied when required. The significance level was set at *α* = 0.05.

Ketone-body results were not included in the inferential analysis because the records were sparse and the original recording format did not permit a reliable distinction between an unrecorded result and a confirmed negative result. These observations were retained in the source dataset and were treated descriptively only.

**Heart-rate monitoring. Heart rate (HR)** was recorded using Polar team monitoring systems during five monitoring weeks (Weeks 1, 3, 5, 7, and 8). Monitoring was conducted over six days during each monitoring week, yielding a total of 30 monitoring days. The recordings represented extended daytime HR monitoring (approximately 08:00–19:00), rather than individual training sessions. Maximum heart rate (HRmax) was estimated for each athlete using the age-predicted formula HRmax = 220 − age. HR intensity was categorized into five individualized zones: 50%–59%, 60%–69%, 70%–79%, 80%–89%, and 90%–100% of HRmax.

For each athlete, daily HR-zone durations were summed within each monitoring week to yield one athlete-level weekly value for each HR zone. Thus, the athlete, rather than the individual monitoring day or training session, constituted the statistical unit (*n* = 8). Because total valid monitored duration varied across monitoring weeks, HR-zone exposure was analyzed both as absolute duration (min) and as a proportion of each athlete's total valid monitored time. Normalized HR-zone exposure was calculated as:

HR-zoneexposure(%)=timeintherespectiveHRzone/totalvalidmonitoredtime×100.

The denominator comprised the entire valid monitored period, including time spent below 50% HRmax. Absolute HR-zone duration was retained as a measure of monitored exposure, whereas normalized HR-zone proportions were used to evaluate relative HR-intensity distribution while accounting for differences in total monitored duration.

**Body composition** was assessed post-training on Saturdays during Weeks 1, 5, and 8 using an InBody 666 body composition analyzer. Participants stood barefoot on the device electrodes and held the hand electrodes with both hands while maintaining a stable upright posture during measurement. Bioelectrical impedance analysis (BIA) was used to assess body mass index (BMI), body fat mass (BFM), body fat percentage (BFR), basal metabolic rate (BMR), and visceral fat level (VFL). BMI, body fat mass, body fat percentage, basal metabolic rate, and visceral fat level were analysed separately using one-way repeated-measures ANOVA, with assessment week (Weeks 1, 5, and 8) as the within-subject factor and the athlete as the statistical unit (*n* = 8).

#### 2.3.2 Psychological indicators

**Rating of Perceived Exertion (RPE)** was assessed twice daily during Weeks 1, 3, 5, 7, and 8 using the Borg CR10 scale (25), with 0 representing no exertion and 10 representing maximal exertion. On training days, ratings were obtained before and after training. Two ratings were also obtained on Sunday recovery days; these represented daily perceived exertion rather than responses to a training session. For correlation analyses, the second daily rating was used: the post-training rating on training days and the second rating on Sundays. Each athlete's mean daily RPE was calculated across all 35 monitoring days. Recorded zeros were retained as actual ratings of no exertion and were not treated as missing observations.

**Recovery and stress** were assessed using the Chinese-language Athlete Training State Monitoring Scale developed by Yan (24). The questionnaire was administered after training on Saturdays during Weeks 1, 3, 5, 7, and 8. Items assessed the frequency of experiences during the preceding three days and nights using seven response options scored from 0 to 6. Emotional Stress, General Well-Being, Fatigue, Self-Regulation, Physical Recovery, and Psychological Exhaustion each comprised six items, with complete-subscale totals ranging from 0 to 36. Self-Efficacy comprised four items, with totals ranging from 0 to 24, and Psychological Fatigue comprised five items, with totals ranging from 0 to 30. The scoring instructions specify summation of the corresponding item scores for complete subscales. For correlation analyses, each athlete's mean subscale score across the five assessments was used.

#### 2.3.3 Statistical analysis

No missing data were present for the variables included in the reported analyses; therefore, no missing-data imputation was required. Recorded RPE scores of zero were retained as valid ratings of no exertion.

Statistical analyses were performed using SPSS version 18.0 (IBM Corp., Armonk, NY, USA) and GraphPad Prism version 10. Data are presented as mean ± standard deviation (SD). Normality was assessed using the Shapiro–Wilk test. The athlete was the statistical unit (*n* = 8), with repeated observations matched within athletes.

Paired-sample t-tests were used to compare blood parameters between Week 1 and Week 8. Cohen's *d_z_* was calculated as the mean within-athlete difference divided by the SD of these differences, with differences defined as Week 8 minus Week 1. Corresponding 95% confidence intervals were reported for the mean paired differences.

For urinary outcomes included in the inferential analyses, two-way repeated-measures ANOVA was used, with monitoring week (Weeks 1, 3, 5, 7, and 8) and sampling condition (Pre-training vs. Post-training) as within-subject factors. Each athlete contributed one aggregated value per monitoring week and sampling condition. The models evaluated the effects of monitoring week, sampling condition, and their interaction. Pre-training samples were collected approximately 15 min before the morning training session, whereas Post-training samples were collected approximately 15 min after the afternoon training session. Thus, sampling condition represented the beginning and end of the daily training period rather than measurements taken before and after a single training session. Sphericity was not assumed for effects involving monitoring week, and Greenhouse–Geisser-corrected degrees of freedom and *p* values were reported. The two-level sampling-condition factor did not require a sphericity correction. KET was retained for descriptive reporting only and excluded from inferential analysis.

BMI, body fat mass, body fat percentage, basal metabolic rate, and visceral fat level were analysed separately using one-way repeated-measures ANOVA, with assessment week (Weeks 1, 5, and 8) as the within-subject factor. Each athlete contributed one observation per outcome at each assessment. Sphericity was not assumed, and Greenhouse–Geisser-corrected degrees of freedom and *p* values were reported for all five outcomes. Body-composition pairwise comparisons, where reported, used Tukey-adjusted *p* values.

Each of the eight subscales of the Chinese-language Athlete Training State Monitoring Scale (1) was analysed separately using one-way repeated-measures ANOVA, with monitoring week (Weeks 1, 3, 5, 7, and 8) as the within-subject factor. Each athlete contributed one subscale total at each assessment. Sphericity was not assumed, and Greenhouse–Geisser-corrected degrees of freedom and *p* values were reported for all eight subscales. No *post hoc* pairwise comparisons were performed for these analyses.

For longitudinal RPE analyses, the seven daily observations within each monitoring week were averaged separately for the first and second daily ratings for each athlete. Sunday recovery-day observations and recorded zeros were retained. This yielded one athlete-level weekly mean for each rating occasion at Weeks 1, 3, 5, 7, and 8. Separate one-way repeated-measures ANOVAs were performed for the first and second daily ratings, with monitoring week as the within-subject factor. Sphericity was not assumed, and Greenhouse–Geisser correction was applied to both analyses. No *post hoc* pairwise comparisons were performed.

For HR-zone outcomes, one-way repeated-measures ANOVA was performed separately for absolute HR-zone duration (min) and normalized HR-zone exposure (% of total valid monitored time), with monitoring week (Weeks 1, 3, 5, 7, and 8) as the within-subject factor. Each athlete contributed one aggregated observation per HR zone and exposure measure at each monitoring week. Sphericity was assessed using Mauchly's test, and Greenhouse–Geisser correction was applied when the assumption was violated. Effect sizes for repeated-measures ANOVA were expressed as partial eta squared (*η*^2^p), calculated as the sum of squares for the effect divided by the sum of that effect's sum of squares and its corresponding error sum of squares.

Pearson correlation analyses examined the associations between the change in circulating irisin concentration (Δirisin; Week 8 − Week 1) and each athlete's mean psychological and perceived-exertion measures across the monitoring period. Psychological means were calculated across the five assessments at Weeks 1, 3, 5, 7, and 8. Mean daily RPE was calculated from the second rating on each of the 35 monitored days: the post-training rating on training days and the second daily rating on Sunday recovery days. Each athlete contributed one observation to each correlation analysis (*n* = 8). These analyses related the change in irisin to individual mean scores rather than to changes in psychological or perceived-exertion scores. The 95% confidence intervals for Pearson's *r* were calculated using Fisher's *r*-to-*z* transformation.

To examine baseline dependence, sensitivity analyses evaluated the associations of individual mean emotional stress with Week 1 and Week 8 irisin separately. The association between Week 1 irisin and Δirisin was also calculated. Participant-level values and sensitivity results are provided in Supplementary Tables S4, S5.

Paired *t*-tests and Pearson correlation tests were two-tailed. Given the exploratory nature of this pilot study and the limited sample size, no overall multiplicity adjustment was applied across outcomes. Omnibus ANOVA, paired *t*-test, and correlation *p* values were therefore interpreted as nominal rather than confirmatory. Tukey-adjusted *p* values for body-composition pairwise comparisons, where reported, were identified separately. Interpretation emphasized effect-size estimates, 95% confidence intervals where available, and the magnitude and consistency of the observed responses. An increased risk of Type I error across the multiple outcomes cannot be excluded, and the findings should be considered exploratory and hypothesis-generating.

Graphs were generated using GraphPad Prism version 10.

## 3 Results

### 3.1 Blood

All eight participants completed the 8-week monitoring period and were included in the reported analyses, with no missing data for the variables analyzed.

#### 3.1.1 Lipid metabolism and irisin

LDL and ApoB levels were higher at Week 8 than at Week 1 (LDL: nominal *p* = 0.028, Cohen's *d_z_* = 0.98; ApoB: nominal *p* = 0.032, Cohen's *d_z_* = 0.95; Figures 2D,F). In contrast, Lp(a) and total cholesterol (TC) levels were lower at Week 8 (Lp(a): nominal *p* = 0.045, Cohen's *d_z_* = −0.86; TC: nominal *p* = 0.003, Cohen's *d_z_* = −1.54; Figures 2A,G).

**Figure 2:**
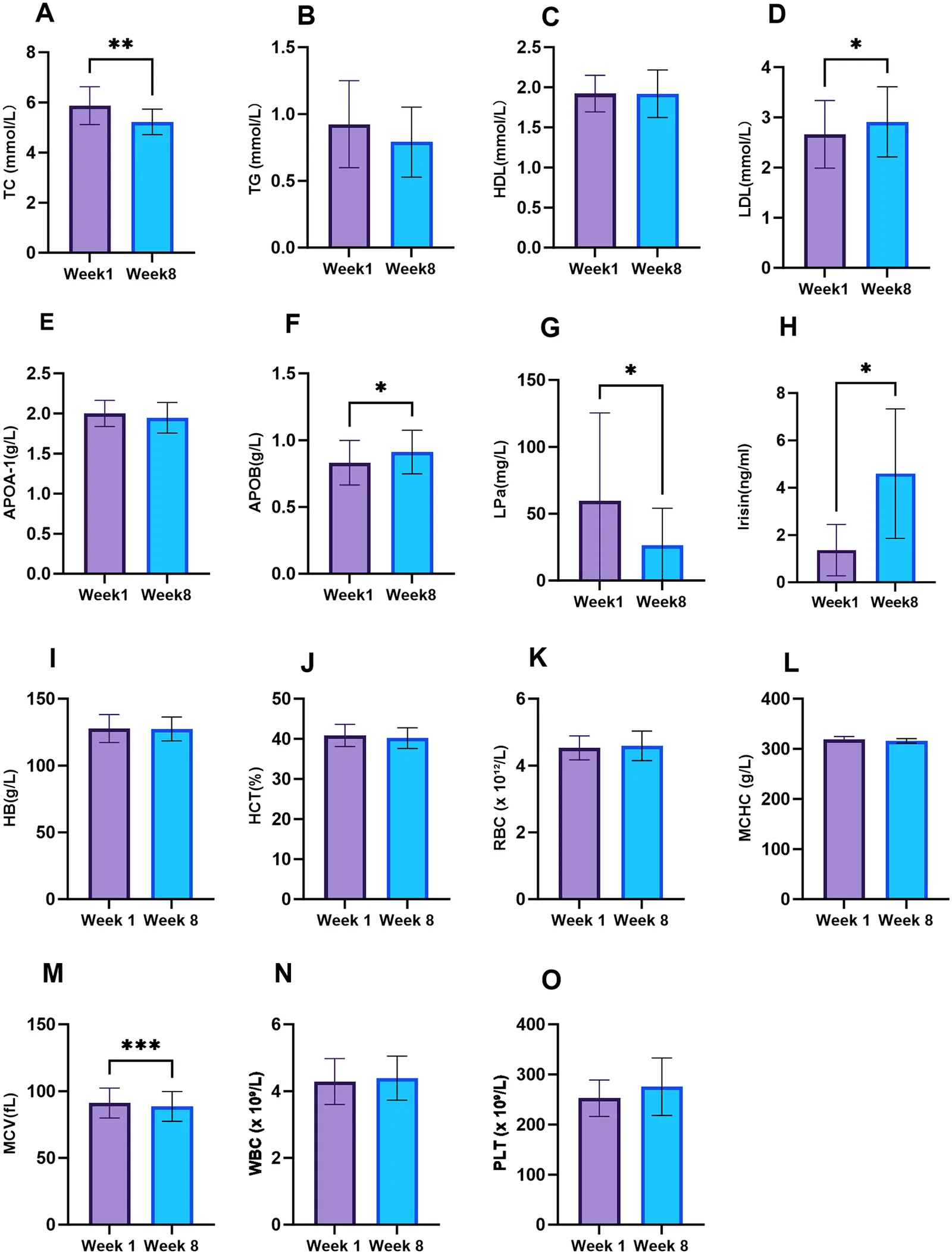
Changes in blood parameters from week 1 to week 8. Data are presented as mean ± SD. Differences between Week 1 and Week 8 were analyzed using paired-sample t-tests. All *p* values are unadjusted and should be interpreted as nominal. *p < 0.05, **p < 0.01, ***p < 0.001. **(a)** TC; **(b)** TG; **(c)** HDL; **(d)** LDL; **(e)** APOA1; **(f)** APOB; **(g)** Lp(a); **(h)** Irisin; **(i)** HB; **(j)** HCT; **(k)** RBC; **(l)** MCHC; **(m)** MCV; **(n)** WBC; **(o)** PLT.

Serum irisin concentrations increased from a mean of 1.357 ng/mL at Week 1 to 4.597 ng/mL at Week 8. The mean paired increase was 3.240 ng/mL (95% CI: 0.338 to 6.143 ng/mL; t(7) = 2.640, nominal *p* = 0.033, Cohen's *d_z_* = 0.93; Figure 2H). Given the known variability of commercial irisin assays, the magnitude of this increase should be interpreted as assay-dependent and descriptive.

#### 3.1.2 Complete blood count (CBC)

MCV decreased from Week 1 to Week 8 (nominal *p* < 0.001, Cohen's *d_z_* = −2.00; Figure 2M), while the observed values remained within the conventional reference interval. Although the standardized within-subject effect size was large, this does not itself indicate a clinically abnormal change. The observed decrease should therefore be interpreted in the context of potential biological and analytical variability. Mean corpuscular hemoglobin concentration (MCHC) values at both time points remained below the reference range of 320 g/L.

### 3.2 Urinalysis and body composition

#### 3.2.1 Urinalysis

PRO showed evidence of variation across the monitoring period (nominal *p* = 0.0052) and between sampling conditions (nominal *p* = 0.0012), with higher post-training values overall; however, the Week × Condition interaction was not statistically significant (*p* = 0.1735). URO showed a marked decrease after Week 1 and evidence of a Week effect (*p* < 0.0001), together with a Week  × Condition interaction (*p* = 0.0335); these findings indicate that the Pre/Post pattern was not uniform across weeks. Urinary pH and SG showed no clear evidence of effects of Week, Condition, or their interaction. LEU also showed no clear evidence of longitudinal or condition-related change. KET was excluded from inferential analysis because of sparse and incompletely interpretable recording and was retained for descriptive purposes only.

#### 3.2.2 Body composition

BMI varied across assessment weeks [F(1.601, 11.21) = 9.219, nominal *p* = 0.0059, *η*^2^*p* = 0.568; Figure 3I]. BFM and BFR showed descriptive decreases from Week 1 to Week 5, but their overall week effects were not statistically significant (BFM: nominal *p* = 0.1407, *η*^2^*p* = 0.253; BFR: nominal *p* = 0.2444, *η*^2^*p* = 0.186; Figures 3G,H). BMR showed a descriptive decrease over the monitoring period, without a statistically significant week effect (nominal *p* = 0.3847, *η*^2^*p* = 0.126; Figure 3K). The week effect for visceral fat level was also not statistically significant (nominal *p* = 0.0992, *η*^2^*p* = 0.281).

**Figure 3:**
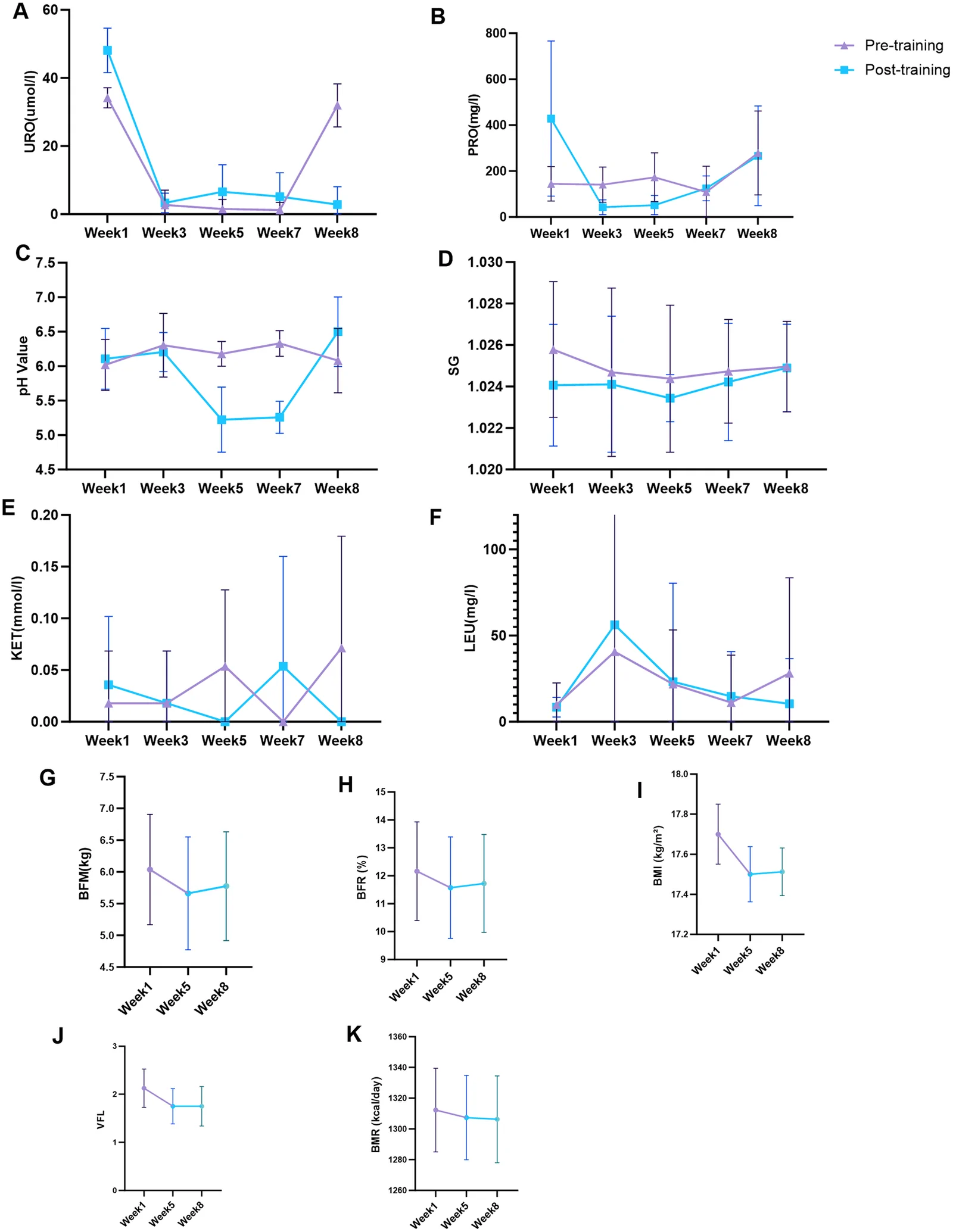
Changes in urine parameters and body composition during 8-week training. Urinary outcomes were assessed under Pre-training (purple triangles) and Post-training (blue squares) sampling conditions at Weeks 1, 3, 5, 7, and 8. Pre-training samples were collected approximately 15 min before the morning training session, whereas Post-training samples were collected approximately 15 min after the afternoon training session. These conditions represent the beginning and end of the daily training period rather than measurements taken before and after a single training session. Body-composition outcomes were assessed at Weeks 1, 5, and 8. Data are presented as mean ± SD across athletes (*n* = 8). KET is presented descriptively and was excluded from inferential analysis. **(A)** URO; **(B)** PRO; **(C)** pH; **(D)** SG; **(E)** KET; **(F)** LEU; **(G)** BFM; **(H)** BFR; **(I)** BMI; **(J)** VFL; **(K)** BMR.

### 3.3 Psychological parameters and heart rate

#### 3.3.1 Chinese-language athlete training state monitoring scale (1)

None of the eight subscales showed a statistically significant monitoring-week effect after Greenhouse–Geisser correction (all nominal *p* ≥ 0.1211; Figures 4A–H). The week effects were F(1.579, 11.05) = 2.662 for self-efficacy (nominal *p* = 0.1211, *η*^2^*p* = 0.276), F(2.004, 14.03) = 1.838 for self-regulation (nominal *p* = 0.1954, *η*^2^*p* = 0.208), and F(1.796, 12.57) = 1.716 for physical recovery (nominal *p* = 0.2198, *η*^2^*p* = 0.197). Complete longitudinal results are provided in Supplementary Table S1.

**Figure 4:**
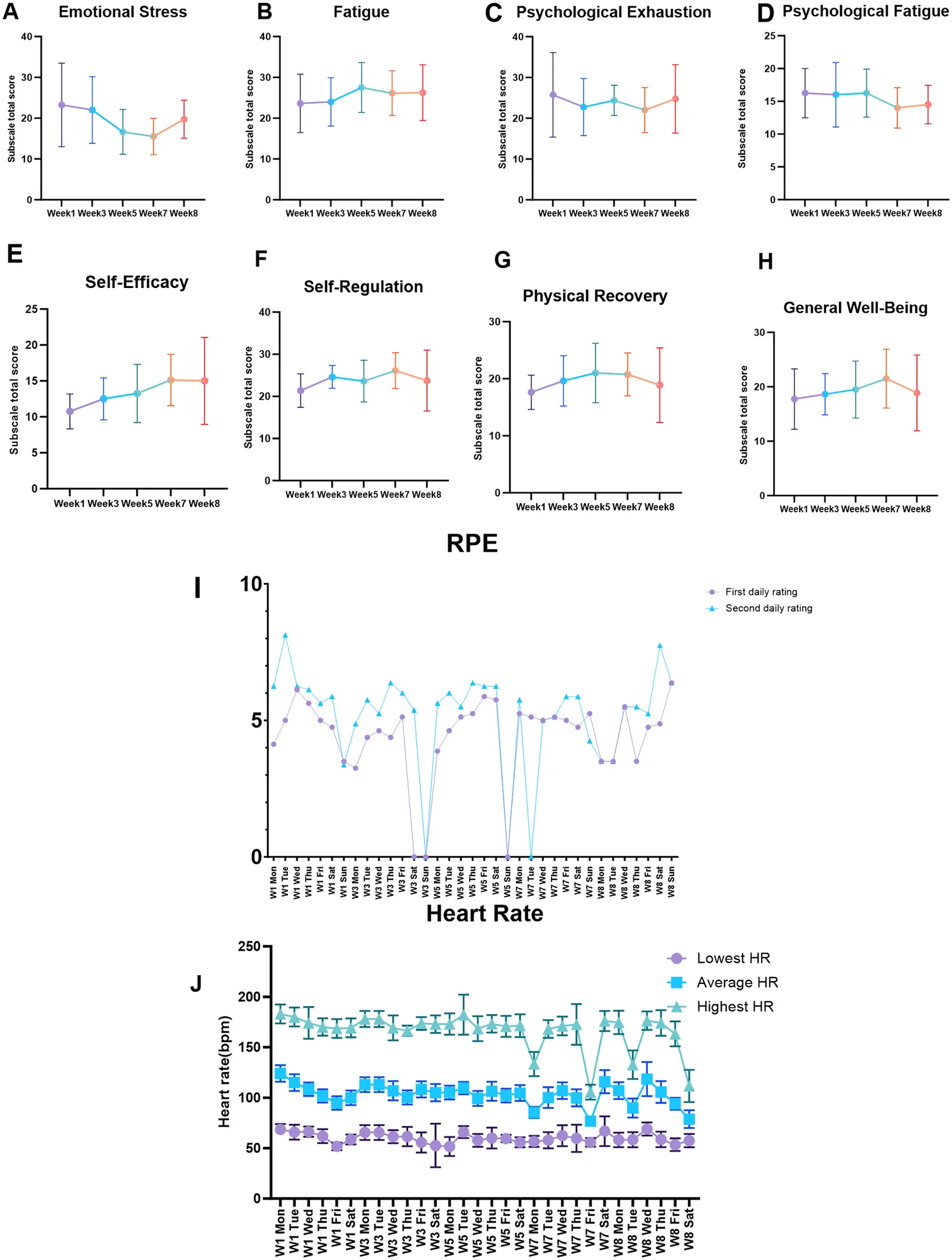
Psychological, perceived-exertion, and heart-rate responses during the 8-week monitoring period. Panels **(A)–(H)** show mean ± SD subscale totals from the Chinese-language Athlete Training State Monitoring Scale (1) across eight athletes at Weeks 1, 3, 5, 7, and 8. Panel I shows the daily mean first and second RPE ratings across athletes over the 35 monitored days; error bars are not displayed. On training days, these corresponded to pre-training and post-training ratings, respectively. On Sunday recovery days, they represented the first and second daily ratings of perceived exertion. Longitudinal RPE analyses used each athlete's weekly mean for each rating occasion, calculated from all seven monitored days, including Sundays and recorded zeros. Panel **(J)** shows mean ± SD lowest, average, and highest heart rates across athletes on the monitored training days. **(A)** Emotional stress; **(B)** fatigue; **(C)** psychological exhaustion; **(D)** psychological fatigue; **(E)** self-efficacy; **(F)** self-regulation; **(G)** physical recovery; **(H)** general well-being; **(I)** RPE; **(J)** heart rate.

#### 3.3.2 RPE

Athlete-level weekly mean RPE varied across monitoring weeks for both the first daily rating [F(2.153, 15.068) = 13.263, nominal *p* < 0.001, *η*^2^*p* = 0.655] and the second daily rating [F(2.071, 14.500) = 12.510, nominal *p* < 0.001, *η*^2^*p* = 0.641], with Greenhouse–Geisser correction applied to both analyses (Figure 4I). On training days, these ratings corresponded to pre-training and post-training assessments, respectively; on Sunday recovery days, they represented the first and second daily assessments of perceived exertion.

#### 3.3.3 Heart rate monitoring

For absolute HR-zone duration, significant monitoring-week effects were observed in the 50%–59% [F(4, 28) = 35.730, nominal *p* < 0.001, *η*^2^*p* = 0.836], 60%–69% [F(1.985, 13.893) = 15.478, nominal *p* < 0.001, *η*^2^*p* = 0.689], and 70%–79% HRmax zones [F(4, 28) = 9.011, nominal *p* < 0.001, *η*^2^*p* = 0.563]. Monitoring-week effects were not statistically significant in the 80%–89% [F(1.633, 11.433) = 4.128, nominal *p* = 0.051, *η*^2^*p* = 0.371] or 90%–100% HRmax zones [F(1.624, 11.369) = 2.551, nominal *p* = 0.128, *η*^2^*p* = 0.267] (Table 4A).

**Table 4A:** Absolute HR-zone duration (min).

HR zone	Week 1	Week 3	Week 5	Week 7	Week 8	F(df)	Nominal *p*	*η* ^2^ *p*
50%–59%	1,074.5 ± 198.9	1,150.0 ± 176.3	988.8 ± 234.0	781.9 ± 174.0	700.9 ± 198.4	F(4,28) = 35.730	<0.001	0.836
60%–69%	612.2 ± 200.2	593.4 ± 253.4	464.5 ± 179.3	438.0 ± 230.9	440.5 ± 249.4	F(1.985,13.893) = 15.478	<0.001	0.689
70%–79%	262.8 ± 90.7	235.0 ± 98.9	201.4 ± 101.1	163.6 ± 96.9	212.2 ± 124.8	F(4,28) = 9.011	<0.001	0.563
80%–89%	68.9 ± 51.9	34.9 ± 30.6	36.5 ± 38.5	41.8 ± 58.2	64.9 ± 79.1	F(1.633,11.433) = 4.128	0.051	0.371
90%–100%	5.9 ± 7.9	0.6 ± 1.8	1.5 ± 3.5	2.9 ± 5.9	5.9 ± 11.5	F(1.624,11.369) = 2.551	0.128	0.267

After HR-zone exposure was normalized to each athlete's total valid monitored time within each monitoring week, only the 50%–59% HRmax zone retained a statistically significant monitoring-week effect [F(4, 28) = 14.131, nominal *p* < 0.001, *η*^2^*p* = 0.669]. No statistically significant monitoring-week effects were observed in the normalized 60%–69% [F(1.855, 12.988) = 3.716, nominal *p* = 0.056, *η*^2^*p* = 0.347], 70%–79% [F(4, 28) = 1.923, nominal *p* = 0.134, *η*^2^*p* = 0.215], 80%–89% [F(1.441, 10.089) = 3.066, nominal *p* = 0.101, *η*^2^*p* = 0.305], or 90%–100% HRmax zones [F(1.525, 10.677) = 2.310, nominal *p* = 0.153, *η*^2^*p* = 0.248] (Table 4B). The corresponding results are also summarized in Supplementary Table S1.

**Table 4B:** Normalized HR-zone exposure (% of total valid monitored time).

HR zone	Week 1	Week 3	Week 5	Week 7	Week 8	F(df)	Nominal *p*	*η* ^2^ *p*
50%–59%	29.46 ± 5.45	30.38 ± 4.66	29.56 ± 6.99	27.71 ± 6.17	21.45 ± 6.07	F(4,28) = 14.131	<0.001	0.669
60%–69%	16.79 ± 5.49	15.68 ± 6.70	13.89 ± 5.36	15.52 ± 8.18	13.48 ± 7.63	F(1.855,12.988) = 3.716	0.056	0.347
70%–79%	7.20 ± 2.49	6.21 ± 2.61	6.02 ± 3.02	5.80 ± 3.43	6.49 ± 3.82	F(4,28) = 1.923	0.134	0.215
80%–89%	1.89 ± 1.42	0.92 ± 0.81	1.09 ± 1.15	1.48 ± 2.06	1.99 ± 2.42	F(1.441,10.089) = 3.066	0.101	0.305
90%–100%	0.16 ± 0.22	0.02 ± 0.05	0.04 ± 0.10	0.10 ± 0.21	0.18 ± 0.35	F(1.525,10.677) = 2.310	0.153	0.248

Values are presented as mean ± SD across athletes (*n* = 8). Absolute HR-zone duration represents the summed athlete-level exposure within each monitoring week. Normalized HR-zone exposure was calculated for each athlete as the time spent in the respective HR zone divided by the total valid monitored time at that monitoring week × 100. The denominator included the entire valid monitored period, including time spent below 50% HRmax. The athlete was the statistical unit. Mauchly's test was used to assess sphericity, and Greenhouse–Geisser corrected degrees of freedom and *p* values are reported where sphericity was violated. All *p* values are nominal and unadjusted for multiple testing. Total valid monitored duration was 3,647 min (60.8 h) at Week 1, 3,785 min (63.1 h) at Week 3, 3,345 min (55.8 h) at Week 5, 2,822 min (47.0 h) at Week 7, and 3,268 min (54.5 h) at Week 8.

### 3.4 Correlation analysis

#### 3.4.1 Irisin and psychological variables

Δirisin was negatively associated with the individual mean emotional-stress score across the monitoring period (*r* = −0.859, 95% CI [−0.974, −0.390], nominal *p* = 0.006). A positive association with individual mean psychological exhaustion was estimated with substantial uncertainty (*r* = 0.704, 95% CI [−0.001, 0.942], nominal *p* = 0.051). The correlation with mean daily RPE was *r* = 0.514 (95% CI [−0.299, 0.895], nominal *p* = 0.193). The remaining correlations are reported in Table 5.

**Table 5:** Pearson correlation coefficients between the change in circulating irisin concentrations (week 8−week 1) and individual mean psychological and load variables across the monitoring period (*n* = 8).

Variable	Pearson's *r*	95% CI	Nominal *p*
RPE	0.514	−0.299 to 0.895	0.193
Emotional stress	−0.859	−0.974 to −0.390	0.006
General well-being	−0.186	−0.787 to 0.597	0.659
Fatigue	−0.031	−0.720 to 0.689	0.942
Self-efficacy	0.302	−0.511 to 0.830	0.467
Self-regulation	0.242	−0.558 to 0.809	0.563
Physical recovery	0.105	−0.647 to 0.754	0.804
Psychological exhaustion	0.704	−0.001 to 0.942	0.051
Psychological fatigue	−0.031	−0.720 to 0.689	0.943

Values represent Pearson's correlation coefficients (r) between the change in circulating irisin concentration (Δirisin; Week 8−Week 1) and each athlete's mean RPE and Chinese-language Athlete Training State Monitoring Scale (1) subscale scores across the monitoring period. For RPE, each athlete's mean was calculated from the second daily rating on each of the 35 monitored days: the post-training rating on training days and the second daily rating on Sunday recovery days. Recorded zeros were retained as valid ratings of no exertion. For psychological subscales, each athlete's mean was calculated across the five assessments at Weeks 1, 3, 5, 7, and 8. Each athlete contributed one observation to each correlation analysis (*n* = 8). The 95% confidence intervals were calculated using Fisher's *r*-to-*z* transformation. All *p* values are two-tailed and unadjusted for multiple testing and should be interpreted as nominal. Given the small sample size and exploratory design, interpretation should emphasize the magnitude and confidence intervals of the correlation estimates. Participant-level values are provided in Supplementary Table S4.

In sensitivity analyses, individual mean emotional stress was positively associated with Week 1 irisin, although its confidence interval included zero (r = 0.676, 95% CI [−0.054, 0.935], nominal *p* = 0.065), and negatively associated with Week 8 irisin (*r* = −0.821, 95% CI [−0.966, −0.275], nominal *p* = 0.013). Week 1 irisin was inversely associated with Δirisin (*r* = −0.761, 95% CI [−0.954, −0.121], nominal *p* = 0.028). The two participants with negative Δirisin had the two highest Week 1 concentrations. Participant-level data and sensitivity results are presented in Supplementary Tables S4 S5. Given the sample size of eight athletes, these correlations should be interpreted as exploratory estimates with substantial uncertainty.

Given the small sample size (*n* = 8), the observed correlation coefficients may be susceptible to magnitude inflation and should therefore be interpreted cautiously. These findings are considered exploratory and hypothesis-generating rather than confirmatory.

## 4 Discussion

This exploratory pilot investigation examined multidimensional physiological, hematological, perceptual, psychological, and irisin responses during an 8-week pre-competition mesocycle in high-level rhythmic gymnasts. The principal findings were that circulating irisin increased from Week 1 to Week 8, MCHC remained below the standard reference range, selected lipid and urinary markers changed across the monitoring period, and exploratory associations between Δ irisin and selected psychological variables were examined. Importantly, several psychological stress-related outcomes showed no clear evidence of longitudinal change, and the present findings should therefore be interpreted with caution.

The observed rise in irisin is consistent with previous evidence demonstrating exercise-responsive myokine secretion and metabolic-hormonal remodeling in response to training stimuli (17–20). However, the physiological and psychological significance of circulating irisin responses in high-level aesthetic sport athletes remains incompletely understood. In the present study, the change in irisin from Week 1 to Week 8 was negatively associated with the individual mean emotional-stress score across the five monitoring assessments, whereas its positive association with individual mean psychological exhaustion was estimated with substantial uncertainty. These analyses concern mean psychological scores across the monitoring period and do not demonstrate that changes in psychological scores accompanied changes in irisin.

Sensitivity analyses showed a positive association of mean emotional stress with Week 1 irisin, with a confidence interval spanning zero, and a negative association with Week 8 irisin. Although the inverse association was also present for the endpoint concentration, this does not establish that the change-score association is independent of baseline differences. Week 1 irisin was inversely associated with Δirisin, and the two participants whose irisin decreased had the highest baseline concentrations. Because Δirisin includes subtraction of the baseline value, mathematical coupling, measurement variability, and regression to the mean may contribute to the observed pattern, although their respective contributions cannot be determined from these analyses. Given the small sample size, observational design, and absence of adjustment for multiple testing, these findings remain exploratory and hypothesis-generating and should not be interpreted as evidence of an irisin-mediated psychological adaptation or buffering effect.

Although MCV decreased over the monitoring period (nominal *p* < 0.001) with a large standardized within-subject effect size, values remained within the conventional reference interval. Therefore, the magnitude of the standardized effect should not be interpreted as evidence of a clinically abnormal hematological change, particularly given the potential contribution of biological and analytical variability. Persistently low MCHC (<320 g/L) may indicate reduced erythrocyte hemoglobinization; however, MCHC alone is insufficient to establish iron deficiency. Hypochromic erythrocyte and reticulocyte indices have been investigated as markers of iron deficiency in female athletes (26). Rhythmic gymnasts have also been reported to be at risk of low energy availability and inadequate micronutrient intake, including iron-related dietary insufficiency (27–29). Nevertheless, because direct nutritional intake, ferritin, transferrin saturation, endocrine markers, and menstrual-cycle data were not collected in the present study, no inference regarding iron deficiency or low energy availability can be made from the current data alone.

The lipid profile showed a mixed pattern, with a decrease in total cholesterol accompanied by increases in LDL and ApoB over the monitoring period. Given the small sample size, this apparently discordant pattern should be interpreted cautiously and should not be considered evidence of a specific training-induced lipid adaptation. It may reflect inter-individual variability, changes in other lipid fractions, or biological and analytical variability across measurements. Further studies with larger cohorts and more comprehensive lipid profiling are required to determine the physiological significance of these changes.

Variations in urine specific gravity may reflect differences in urine concentration and hydration status, whereas exercise-induced proteinuria is a recognized transient response following strenuous exercise (30, 31). In the present study, SG did not show clear evidence of change across monitoring weeks or sampling conditions, while PRO showed variation across weeks and higher post-training values overall. URO also varied across the monitoring period, with the Pre/Post pattern differing between weeks. These findings should be interpreted cautiously, particularly because sampling timing, hydration status, dietary factors, and the semi-quantitative nature of some dipstick measures may influence urinary values. Urine specific gravity remains a practical hydration-related measure in athletic settings (32, 33), while urine-based biomarkers more broadly provide a non-invasive approach for monitoring exercise-related physiological perturbations (34, 35). However, the present results do not by themselves demonstrate a longitudinal change in hydration status. In the present study, absolute HR-zone durations varied across monitoring weeks in several HR-intensity zones; however, after normalization to total valid monitored time, only the 50%–59% HRmax zone retained a statistically significant monitoring-week effect. This distinction indicates that part of the temporal variation in absolute HR-zone duration reflected differences in total monitored duration rather than a systematic change in relative HR-intensity distribution, and supports the separate interpretation of monitored exposure and relative HR-intensity distribution in future monitoring studies.

An integrated monitoring approach in rhythmic gymnastics may include subjective measures such as RPE and Chinese-language Athlete Training State Monitoring Scale (1), periodic hematological screening when clinically indicated, simple urine-based hydration checks, and body composition monitoring when interpreted cautiously. Subjective self-report measures remain valuable components of athlete monitoring systems (36), particularly when integrated with physiological and perceptual indicators.

This study has several limitations. First, the extremely small sample size and single-team design substantially limit statistical power and generalizability. Second, multiple statistical comparisons were performed across the physiological, hematological, perceptual, psychological, and correlation analyses. Although Tukey adjustment was used for body-composition pairwise comparisons where reported, no overall multiplicity adjustment was applied across outcomes. Omnibus ANOVA, paired t-test, and correlation *p* values should therefore be regarded as nominal, and an increased risk of Type I error cannot be excluded. Interpretation should emphasize effect-size estimates, confidence intervals where available, and the magnitude and consistency of the observed responses. The findings should be considered exploratory and hypothesis-generating rather than confirmatory. Third, the observational design without a control group prevents causal interpretation of the observed physiological and psychological changes. Fourth, important potential confounding variables, including menstrual cycle status, nutritional intake, sleep quality, hydration standardization, and psychosocial stress outside sport, were not systematically controlled. Fifth, the limited number of sampling time points constrains interpretation of adaptation trajectories across the entire pre-competition period. Sixth, body composition was assessed using bioelectrical impedance analysis, which may be influenced by hydration status. Seventh, methodological limitations associated with circulating irisin quantification should be acknowledged. Back-calculation of the nominal calibration standards from the second-order polynomial equation showed appreciable discrepancies in parts of the lower calibration range (approximately +31% at 78.13 pg/mL, −11% at 312.5 pg/mL, and −13% at 625 pg/mL on the on-plate scale), whereas agreement was substantially closer at the higher standards. Because several samples, particularly at Week 1, were located within the lower concentration range, differential calibration error across the assay range cannot be excluded and may have influenced the estimated magnitude of the Week 1-to-Week 8 change in circulating irisin. Eighth, external load indicators such as apparatus repetitions, landing counts, jump counts, and direct performance outcomes were not systematically collected. Finally, heart rate was assessed using extended daytime monitoring rather than standardized session-specific recordings, and total valid monitored duration varied across monitoring weeks. Although HR-zone exposure was additionally normalized to total valid monitored time to account for differences in monitoring coverage, this variability should be considered when interpreting the absolute HR-zone durations. Furthermore, HRmax was estimated using the age-predicted formula (220 − age) rather than directly measured using a maximal exercise test, which may have introduced individual error in HR-zone classification.

Future studies should incorporate larger cohorts, controlled comparison groups, repeated mechanistic measurements, nutritional assessment, endocrine profiling, external workload quantification, neuromuscular testing, and direct performance outcomes. Such approaches would help determine whether multidimensional monitoring can support individualized training adjustment, nutritional intervention, and recovery management in elite aesthetic sports.

## 5 Conclusion

This exploratory pilot study suggests that multidimensional longitudinal monitoring may provide useful insight into physiological, hematological, perceptual, and psychological responses during intensified pre-competition training in elite rhythmic gymnasts. Although circulating irisin concentrations increased during the intervention period, the functional significance of this response remains unclear. The coexistence of altered physiological markers and exploratory relationships with selected psychological variables highlights the complexity of adaptation in aesthetic sports and supports the need for cautious, integrated athlete monitoring approaches in future research and practice.

Detailed statistical outcomes, effect sizes, and confidence intervals are presented in Supplementary Table S1.

## Data Availability

The raw data supporting the conclusions of this article will be made available by the authors, without undue reservation.
