# Supplementary material for: Exploratory longitudinal monitoring of physiological, hematological, psychological, and irisin responses during an 8-week pre-competition period in elite rhythmic gymnasts
Source: Front Sports Act Living. 2026 Oct 21;8:1925319. doi: 10.3389/fspor.2026.1925319 (PMC13731363; doi:10.3389/fspor.2026.1925319)
Supplement: Supplementary file 1 [file Datasheet1.pdf]

STROBE Statement - Completed Checklist

Exploratory Longitudinal Monitoring of Physiological, Hematological, Psychological, and Irisin Responses During an 8-Week Pre-Competition Period in Elite Rhythmic Gymnasts

Note: This checklist is aligned with the revised manuscript. All eight participants completed the 8-week monitoring period and no missing data were present for the variables included in the reported analyses. Page numbers/section locations correspond to the revised manuscript and should be interpreted together with the manuscript headings.

Section	Item	STROBE recommendation	Location in manuscript	Completed response
Title and abstract	1(a)	Indicate the study design with a commonly used term in the title or abstract.	Title; Abstract	The title identifies longitudinal monitoring and the Abstract identifies the work as an exploratory pilot study over an 8-week pre-competition period.
Title and abstract	1(b)	Provide an informative and balanced summary of what was done and what was found.	Abstract	Reports n=8, monitoring duration, multidimensional measurements, principal findings, and cautious interpretation.
Introduction	2	Explain the scientific background and rationale for the investigation.	Introduction	Describes rhythmic-gymnastics training demands, multidimensional monitoring, recovery/stress assessment, and rationale for exploratory irisin monitoring.
Introduction	3	State specific objectives, including any prespecified hypotheses.	Introduction, final paragraph	States the study aim and exploratory hypothesis; no specific mechanistic hypothesis regarding irisin-mediated psychological adaptation was proposed.
Methods	4	Present key elements of study design early in the paper.	Sections 2.1-2.2	Exploratory longitudinal pilot monitoring of eight rhythmic gymnasts during an 8-week pre-competition period.
Methods	5	Describe setting, locations, and relevant dates/periods of recruitment, exposure, follow-up, and data collection.	Section 2.2; Figure 1	High-level competitive team in China; pre-competition period commenced in July and lasted 8 weeks; assessment schedule shown in Figure 1.
Methods	6(a)	Give eligibility criteria, sources and methods of participant selection, and methods of follow-up.	Section 2.2	The study cohort comprised the eight athletes participating in the monitored pre-competition training program. All eight athletes held a nationally recognized elite athlete classification in China. No additional selection based on physiological, hematological, body-composition, or psychological characteristics was applied.
Methods	6(b)	For matched cohort studies, give matching criteria and numbers of exposed/unexposed.	N/A	Not applicable: single-cohort longitudinal monitoring study without matched exposed/unexposed groups.
Methods	7	Clearly define outcomes, exposures, predictors, potential confounders, and effect modifiers.	Sections 2.3.1-2.3.3	Physiological, hematological, body-composition, RPE, Chinese-language Athlete Training State Monitoring Scale (Yan, 2003), heart-rate, urinalysis and irisin variables are defined; potential confounders are acknowledged.
Methods	8	For each variable, give data sources and details of assessment methods.	Sections 2.3.1-2.3.2	Timing and procedures are described for blood/CBC/lipids/irisin, urinalysis, heart rate, body composition, RPE, and Chinese-language Athlete Training State Monitoring Scale (Yan, 2003).
Methods	9	Describe efforts to address potential sources of bias.	Sections 2.3.1-2.3.3; Discussion limitations	Standardized assessment timing/conditions are reported where

				applicable, and uncontrolled confounding and measurement limitations are acknowledged.
Methods	10	Explain how study size was arrived at.	Section 2.2	The sample size was determined by the number of athletes available in the monitored training squad during the study period; therefore, no formal a priori sample-size calculation was performed.
Methods	11	Explain how quantitative variables were handled in analyses, including groupings if applicable.	Sections 2.3.1-2.3.3	Continuous variables summarized as mean ± SD; HR intensity categorized into five HRmax zones; athlete-level longitudinal means/change scores used as specified for correlations.
Methods	12(a)	Describe all statistical methods, including methods used to control for confounding.	Section 2.3.3	Shapiro-Wilk, Mauchly/Greenhouse-Geisser, paired t-tests, repeated-measures ANOVA, effect sizes, confidence intervals, and Pearson correlations are described; no confounder-adjusted models were performed.
Methods	12(b)	Describe methods used to examine subgroups and interactions.	Section 2.3.3	Two-way repeated-measures ANOVA examined the effects of monitoring week, sampling condition (Pre-training vs. Post-training), and their interaction for urinary variables. No subgroup analyses were performed.
Methods	12(c)	Explain how missing data were addressed.	Section 2.3.3	No missing data were present for the variables included in the reported analyses; therefore, no missing-data imputation was required.
Methods	12(d)	Explain how loss to follow-up was addressed.	Results opening paragraph	All eight participants completed the 8-week monitoring period and were included in the reported analyses; therefore, there was no loss to follow-up.
Methods	12(e)	Describe any sensitivity analyses.	Section 2.3.3; Supplementary Table S5	Baseline-dependence sensitivity analyses examined the associations of individual mean emotional-stress scores with Week 1 and Week 8 irisin concentrations separately, and the association between Week 1 irisin and the change in irisin (Week 8 − Week 1).
Results	13(a)	Report numbers at each stage of the study.	Results opening paragraph	All eight participants completed the 8-week monitoring period and were included in the reported analyses.
Results	13(b)	Give reasons for non-participation at each stage.	N/A	No participant attrition or exclusion occurred during the reported monitoring period.
Results	13(c)	Consider use of a flow diagram.	N/A; Figure 1	A recruitment flow diagram was not necessary for this single-team cohort; Figure 1 presents the longitudinal assessment schedule.
Results	14(a)	Give characteristics of study participants and information on exposures/potential confounders.	Table 1; Sections 2.2-2.2.1	Table 1 reports participant characteristics; training structure is described in the Methods and Tables 2-3.
Results	14(b)	Indicate number of participants with missing data for each variable.	Results opening paragraph	No missing data were present for the variables analyzed; n = 8 throughout the reported analyses.
Results	14(c)	For cohort studies, summarize follow-up	Section 2.2; Figure 1	All athletes were monitored across the

		time.		8-week pre-competition period; repeated-assessment timing is shown in Figure 1.
Results	15	Report outcome events or summary measures over time.	Sections 3.1-3.4; Tables 4-5; Figures 2-4; Supplementary Table S1	Longitudinal outcomes are reported for blood/irisin, urinalysis, body composition, Chinese-language Athlete Training State Monitoring Scale (Yan, 2003), RPE, and heart-rate variables.
Results	16(a)	Give unadjusted and, if applicable, adjusted estimates and their precision.	Sections 3.1-3.4; Table 5; Supplementary Table S1	Unadjusted estimates are reported; effect sizes and 95% CIs are provided for principal paired comparisons and Pearson correlations; no adjusted estimates were calculated.
Results	16(b)	Report category boundaries when continuous variables were categorized.	Section 2.3.1; Table 4	HR zones defined as 50-59%, 60-69%, 70-79%, 80-89%, and 90-100% of estimated HRmax.
Results	16(c)	If relevant, translate relative risk estimates into absolute risk.	N/A	Not applicable: no relative-risk analyses were performed.
Results	17	Report other analyses, such as subgroup, interaction, and sensitivity analyses.	Section 3.4; Supplementary Table S5	Results of the baseline-dependence sensitivity analyses include the associations of individual mean emotional-stress scores with Week 1 and Week 8 irisin concentrations separately, and the association between Week 1 irisin and the change in irisin (Week 8 − Week 1).
Discussion	18	Summarize key results with reference to study objectives.	Discussion	Summarizes multidimensional physiological, hematological, perceptual, psychological, and irisin findings in relation to the exploratory monitoring objective.
Discussion	19	Discuss limitations, including sources of potential bias or imprecision.	Discussion, limitations paragraph	Addresses very small sample/single-team design, multiplicity, lack of control group, uncontrolled confounders, limited sampling, BIA, irisin assay variability, external-load limitations, and estimated HRmax.
Discussion	20	Give a cautious overall interpretation considering objectives, limitations, multiplicity, and other evidence.	Discussion; Conclusion	Interpretation emphasizes exploratory/hypothesis-generating findings, uncertainty, potential Type I error, assay dependence, and absence of causal inference.
Discussion	21	Discuss generalisability/external validity.	Discussion, limitations/future studies	Small single-team sample is explicitly stated to limit generalizability; larger controlled cohorts are recommended.
Other information	22	Give source of funding and role of funders.	Funding statement	The authors declare that no financial support was received for the research, authorship, and/or publication of this article.
