# Supplementary material for: Exploratory longitudinal monitoring of physiological, hematological, psychological, and irisin responses during an 8-week pre-competition period in elite rhythmic gymnasts
Source: Front Sports Act Living. 2026 Oct 21;8:1925319. doi: 10.3389/fspor.2026.1925319 (PMC13731363; doi:10.3389/fspor.2026.1925319)
Supplement: Supplementary file 2 [file Table1.docx]

**Supplementary Statistical Table**

| Variable | Statistical Test | p-value | Effect Size | 95% CI |
| --- | --- | --- | --- | --- |
| Irisin | Paired t-test | 0.033 | dz= 0.93 | 0.338 to 6.143 ng/mL |
| TC | Paired t-test | 0.003 | dz = −1.54 | −0.995 to −0.295 mmol/L |
| LDL | Paired t-test | 0.028 | dz = 0.98 | 0.037 to 0.461 mmol/L |
| APOB | Paired t-test | 0.032 | dz = 0.95 | 0.009 to 0.151 g/L |
| Lp(a) | Paired t-test | 0.045 | dz = −0.86 | −65.321 to −0.979 mg/L |
| MCV | Paired t-test | <0.001 | dz = −2.00 | −3.491 to −1.434 fL |
| BMI | Repeated-measures ANOVA | 0.0059 | η²p = 0.568 | — |
| BFM | F(1.691, 11.83)=2.373 | 0.1407 | η²p =0.253 |  |
| BFR | F(1.437, 10.06)=1.600 | 0.2444 | η²p =0.186 |  |
| VFL | F(1.996, 13.97)=2.739 | 0.0992 | η²p =0.281 |  |
| BMR | F(1.781, 12.47)=1.005 | 0.3847 | η²p =0.126 |  |
| Emotional Stress | Repeated-measures ANOVA (GG-corrected) | 0.1913 | η²p = 0.208 | — |
| Fatigue | Repeated-measures ANOVA (GG-corrected) | 0.2833 | η²p = 0.164 | — |
| Psychological Exhaustion | Repeated-measures ANOVA (GG-corrected) | 0.6773 | η²p = 0.060 | — |
| Psychological Fatigue | Repeated-measures ANOVA (GG-corrected) | 0.4333 | η²p = 0.120 | — |
| General Well-Being | Repeated-measures ANOVA (GG-corrected) | 0.601 | η²p = 0.070 | — |
| Self-Efficacy | Repeated-measures ANOVA (GG-corrected) | 0.1211 | η²p = 0.276 | — |
| Self-Regulation | Repeated-measures ANOVA (GG-corrected) | 0.1954 | η²p = 0.208 | — |
| Physical Recovery | Repeated-measures ANOVA (GG-corrected) | 0.2198 | η²p = 0.197 | — |
| HR zone (50–59% HRmax): absolute duration | Repeated-measures ANOVA | <0.001 | η²p = 0.836 | — |
| HR zone (60–69% HRmax): absolute duration | Repeated-measures ANOVA (GG-corrected) | <0.001 | η²p = 0.689 | — |
| HR zone (70–79% HRmax): absolute duration | Repeated-measures ANOVA | <0.001 | η²p = 0.563 | — |
| HR zone (80–89% HRmax): absolute duration | Repeated-measures ANOVA (GG-corrected) | 0.051 | η²p = 0.371 | — |
| HR zone (90–100% HRmax): absolute duration | Repeated-measures ANOVA (GG-corrected) | 0.128 | η²p = 0.267 | — |
| HR zone (50–59% HRmax): normalized exposure | Repeated-measures ANOVA | <0.001 | η²p = 0.669 | — |
| HR zone (60–69% HRmax): normalized exposure | Repeated-measures ANOVA (GG-corrected) | 0.056 | η²p = 0.347 | — |
| HR zone (70–79% HRmax): normalized exposure | Repeated-measures ANOVA | 0.134 | η²p = 0.215 | — |
| HR zone (80–89% HRmax): normalized exposure | Repeated-measures ANOVA (GG-corrected) | 0.101 | η²p = 0.305 | — |
| HR zone (90–100% HRmax): normalized exposure | Repeated-measures ANOVA (GG-corrected) | 0.153 | η²p = 0.248 | — |
| RPE: first daily rating | Repeated-measures ANOVA (GG-corrected) | <0.001 | η²p = 0.655 |  |
| RPE: second daily rating | Repeated-measures ANOVA (GG-corrected) | <0.001 | η²p = 0.641 |  |

**Supplementary Table S1.** Statistical outcomes, effect sizes, and confidence intervals for primary physiological and psychological analyses.

Note. All p values in this table are unadjusted for multiple testing across outcomes and should be interpreted as nominal. Confidence intervals for paired t-tests refer to the mean paired difference (Week 8 − Week 1). Cohen’s dz was used for paired t-tests, and partial eta squared (η²p) was used for repeated-measures ANOVA. The athlete was the statistical unit (n = 8). Body-composition analyses used assessment week (Weeks 1, 5, and 8) as the within-subject factor. Psychological subscale, RPE, and HR-zone analyses used monitoring week (Weeks 1, 3, 5, 7, and 8) as the within-subject factor. For each RPE rating occasion, each athlete’s weekly mean included all seven monitored days, including Sundays and recorded zeros. GG indicates Greenhouse–Geisser correction, which was applied to all body-composition, psychological subscale, and RPE analyses, and to HR-zone analyses where sphericity was violated. Absolute HR-zone duration is expressed in minutes. Normalized exposure was calculated as absolute duration divided by each athlete’s total valid monitored time within the corresponding week × 100, including time below 50% HRmax in the denominator. HR-zone p values and effect sizes correspond to Table 4A for absolute duration and Table 4B for normalized exposure. A dash indicates that a confidence interval is not reported.

| **Time point** | **Participant** | **OD-A** | **OD-B** | **Mean OD** | **Corrected OD*** | **On-plate conc. (pg/mL)** | **Serum conc. (pg/mL, ×2)** | **CV (%)** |
| --- | --- | --- | --- | --- | --- | --- | --- | --- |
| Week 8 | A | 0.782 | 0.902 | 0.842 | 0.780 | 1074.77 | 2149.55 | 10.06 |
|  | B | 1.802 | 2.119 | 1.961 | 1.899 | 3395.17 | 6790.35 | 11.44 |
|  | C | 1.202 | 1.362 | 1.282 | 1.220 | 1868.77 | 3737.53 | 8.84 |
|  | D | 1.956 | 2.187 | 2.071 | 2.009 | 3677.58 | 7355.16 | 7.89 |
|  | E | 1.727 | 2.003 | 1.865 | 1.803 | 3157.83 | 6315.67 | 10.49 |
|  | F | 0.726 | 0.795 | 0.760 | 0.698 | 943.80 | 1887.61 | 6.38 |
|  | G | 0.377 | 0.446 | 0.412 | 0.350 | 445.49 | 890.98 | 11.90 |
|  | H | 2.003 | 2.251 | 2.127 | 2.065 | 3824.48 | 7648.97 | 8.22 |
| Week 1 | A | 0.277 | 0.330 | 0.303 | 0.241 | 310.13 | 620.26 | 12.42 |
|  | B | 0.658 | 0.723 | 0.690 | 0.628 | 835.93 | 1671.87 | 6.65 |
|  | C | 0.215 | 0.238 | 0.227 | 0.165 | 219.95 | 439.90 | 7.02 |
|  | D | 0.235 | 0.260 | 0.248 | 0.186 | 244.04 | 488.09 | 7.24 |
|  | E | 0.334 | 0.395 | 0.364 | 0.302 | 384.77 | 769.55 | 11.82 |
|  | F | 1.102 | 1.197 | 1.150 | 1.088 | 1613.78 | 3227.57 | 5.87 |
|  | G | 0.925 | 1.115 | 1.020 | 0.958 | 1377.02 | 2754.03 | 13.15 |
|  | H | 0.380 | 0.436 | 0.408 | 0.346 | 440.49 | 880.98 | 9.81 |

**Supplementary Table S2.** Individual sample concentrations and duplicate optical-density (OD) results.

Note: *Corrected OD = mean OD − blank OD (0.062). Duplicate CV (%) = standard deviation of the two replicate OD readings divided by their mean, expressed as a percentage. Duplicate CVs are reported descriptively as measures of within-pair agreement. Because OD-B exceeded OD-A across all 16 sample pairs, the between-replicate differences should not be interpreted as representing random analytical imprecision alone. On-plate concentrations were calculated using the second-order polynomial calibration equation reported in Supplementary Table S3; serum concentrations = on-plate concentration × 2 (dilution factor). Participant codes A–H represent the same eight athletes at Week 1 and Week 8. All samples were analyzed on the same ELISA plate using the same calibration relationship.

| **Calibrator (pg/mL)** | **Raw OD** | **Blank-Corrected OD*** |
| --- | --- | --- |
| 5000 | 2.539 | 2.477 |
| 2500 | 1.604 | 1.542 |
| 1250 | 0.953 | 0.891 |
| 625 | 0.485 | 0.423 |
| 312.5 | 0.276 | 0.214 |
| 156.25 | 0.180 | 0.118 |
| 78.13 | 0.120 | 0.058 |
| 0 (blank) | 0.062 | 0 |

**Supplementary Table S3.** Calibration data used for serum irisin concentration calculation

Calibration standards were assayed as single wells rather than in duplicate; therefore, duplicate calibrator OD measurements were not generated. The blank OD (0.062) was subtracted from each calibrator OD value. The original assay calculation record used the second-order polynomial equation y = 395.8x² + 1014.3x + 42.208 (R² = 0.9994), where x represents the blank-corrected OD and y represents the calculated on-plate concentration (pg/mL). The same calibration relationship was used for all Week 1 and Week 8 serum samples analyzed on the same ELISA plate

| **Participant** | **Week 1 irisin (pg/mL)** | **Week 8 irisin (pg/mL)** | **Δirisin (pg/mL)** | **Mean daily RPE** |
| --- | --- | --- | --- | --- |
| A | 620.26 | 2149.55 | 1529.29 | 5.514286 |
| B | 1671.87 | 6790.35 | 5118.48 | 5.314286 |
| C | 439.90 | 3737.53 | 3297.63 | 4.828571 |
| D | 488.09 | 7355.16 | 6867.07 | 5.771429 |
| E | 769.55 | 6315.67 | 5546.12 | 4.942857 |
| F | 3227.57 | 1887.61 | −1339.96 | 5.085714 |
| G | 2754.03 | 890.98 | −1863.05 | 4.600000 |
| H | 880.98 | 7648.97 | 6767.99 | 5.200000 |

**Supplementary Table S4.** Participant-level irisin concentrations and mean perceived-exertion and psychological scores used in the correlation analyses.

Panel A. Serum irisin concentrations and mean daily RPE.

| **Participant** | **ES** | **GWB** | **FAT** | **SE** | **SR** | **PR** | **PE** | **PF** |
| --- | --- | --- | --- | --- | --- | --- | --- | --- |
| A | 20.80 | 20.00 | 26.60 | 12.60 | 20.40 | 18.00 | 21.80 | 18.40 |
| B | 19.40 | 17.60 | 28.40 | 15.00 | 23.80 | 17.00 | 27.00 | 18.00 |
| C | 23.20 | 23.00 | 33.80 | 19.20 | 31.20 | 28.80 | 24.40 | 16.80 |
| D | 16.40 | 18.00 | 24.20 | 13.60 | 25.20 | 18.20 | 22.00 | 14.60 |
| E | 20.60 | 21.00 | 28.60 | 11.60 | 26.00 | 20.20 | 32.40 | 16.20 |
| F | 27.00 | 21.40 | 21.40 | 12.60 | 23.20 | 19.20 | 18.60 | 15.20 |
| G | 23.20 | 16.60 | 24.80 | 9.60 | 21.00 | 16.40 | 19.20 | 12.80 |
| H | 16.80 | 16.40 | 16.20 | 12.40 | 20.40 | 18.80 | 26.00 | 11.20 |

Panel B. Individual mean psychological subscale scores across the five monitoring assessments.

**Note**: Participant codes A–H identify the same eight athletes as in Supplementary Table S2. Serum irisin concentrations reproduce the dilution-corrected values reported in that table. Δirisin was calculated as Week 8 minus Week 1. Mean daily RPE was calculated from the second rating on each of the 35 monitoring days across Weeks 1, 3, 5, 7, and 8: the post-training rating on training days and the second daily rating on Sunday recovery days. Recorded zeros were retained as actual ratings of no exertion. RPE means are presented to six decimal places; analyses used unrounded means. Psychological values represent each athlete’s mean subscale score across the five assessments at Weeks 1, 3, 5, 7, and 8, rather than individual-item ratings or changes in psychological scores. Each athlete contributed one observation to each correlation analysis (n = 8).

**Abbreviations**: RPE, rating of perceived exertion; ES, emotional stress; GWB, general well-being; FAT, fatigue; SE, self-efficacy; SR, self-regulation; PR, physical recovery; PE, psychological exhaustion; PF, psychological fatigue.

| **Variable pair** | **Pearson’s r** | **95% CI** | **Nominal p** |
| --- | --- | --- | --- |
| Mean emotional stress vs. Week 1 irisin | 0.676 | −0.054 to 0.935 | 0.065 |
| Mean emotional stress vs. Week 8 irisin | −0.821 | −0.966 to −0.275 | 0.013 |
| Mean emotional stress vs. Δirisin | −0.859 | −0.974 to −0.390 | 0.006 |
| Week 1 irisin vs. Δirisin | −0.761 | −0.954 to −0.121 | 0.028 |

**Supplementary Table S5. Sensitivity analyses examining baseline dependence of the irisin–mean emotional-stress association.**

Note: Δirisin = Week 8 − Week 1. Mean emotional stress represents each athlete’s mean score across Weeks 1, 3, 5, 7, and 8. Each analysis included eight athletes. All p values are two-tailed and unadjusted for multiple testing. Confidence intervals were calculated using Fisher’s r-to-z transformation.
